# Editorial: Somatic Cell Gene Editing for Treating Diseases

**DOI:** 10.3389/fcell.2021.828195

**Published:** 2021-12-23

**Authors:** Raymond Ching-Bong Wong, Junjiu Huang, Dali Li, Olga Amaral

**Affiliations:** ^1^ Centre for Eye Research Australia, Royal Victorian Eye and Ear Hospital, Melbourne, VIC, Australia; ^2^ Ophthalmology, Department of Surgery, University of Melbourne, Melbourne, VIC, Australia; ^3^ Shenzhen Eye Hospital, Shenzhen University School of Medicine, Shenzhen, China; ^4^ MOE Key Laboratory of Gene Function and Regulation, State Key Laboratory of Biocontrol, School of Life Sciences, Sun Yat-sen University, Guangzhou, China; ^5^ Key Laboratory of Reproductive Medicine of Guangdong Province, the First Affiliated Hospital and School of Life Sciences, Sun Yat-sen University, Guangzhou, China; ^6^ Shanghai Frontiers Science Center of Genome Editing and Cell Therapy, Shanghai Key Laboratory of Regulatory Biology, Institute of Biomedical Sciences and School of Life Sciences, East China Normal University, Shanghai, China; ^7^ Departamento de Genética Humana, Unidade de Investigação e Desenvolvimento, Instituto Nacional de Saúde Dr Ricardo Jorge (INSA), Porto, Portugal; ^8^ CECA, ICETA, University of Porto, Porto, Portugal

**Keywords:** gene therapy, human genetic diseases, viral infectious diseases, degenerative diseases, CRISPR/Cas, off-targets

Recent breakthroughs in genetic engineering allow us to precisely edit the genome with high efficiency, which can be achieved using various genomic tools including meganucleases, zinc-finger nucleases (ZFNs), transcription activator-like effector nucleases (TALENs) and clustered regularly interspaced short palindromic repeats (CRISPR)-associated (Cas) proteins. These tools can be engineered to recognize and induce DNA or RNA cleavage at target sites to generate sequence editing. In recent years, a range of CRISPR-based tools have been developed that enable different molecular functions. For instance, precise targeted DNA editing can be performed using base editors or prime editors; an inactive-Cas9 can be coupled with transcriptional activators for gene activation (CRISPR activation), or coupled with transcriptional repressors for gene repression (CRISPR inhibition). In addition, RNA can be targeted for editing using base editors, or targeted for knockdown using Cas13. These genomic tools enable faster, more accurate and more efficient gene modification, providing an exciting therapeutic approach for somatic cell gene editing to treat diseases.

The ability to precisely correct disease-causing mutations would be critical to the development of gene therapy for inherited diseases. Abdelnour et al. discussed the potential of CRISPR/Cas9 for gene editing to treat inherited diseases. The authors reviewed recent discoveries on the uses of CRISPR/Cas9 for rectifying genetic diseases, and highlighted the versatility of gene editing as a therapeutic approach and its limitations. Also, Vicente et al. discussed the current hurdles of using CRISPR/Cas for gene editing and reviewed the approaches to reduce off-target effects. Off-target effects are a major concern in gene editing and the authors explored and revised off-targets from various points of view. Moreover, their thorough revision also included new strategies to help overcome issues related to off-targets.

Beyond inherited diseases, genome editing can also be applied to cancer immunotherapy and viral infectious diseases. Ou et al. reviewed the current advances in using CRISPR/Cas for developing chimeric antigen receptor T (CAR-T) and T cell receptor T(TCR-T) to target cancer cells. The authors provided a discerning overview of the CRISPR-Cas9-based technology and included diverse aspects regarding its innovative application in the field of cancer research and therapy. Furthermore, Zhang et al. reviewed the use of genome editing technologies as cellular defence against viral pathogens. In particular, the authors highlighted gene editing strategies as a novel approach to treat viral infections, including HIV, HBV, HPV, HSV and SARS-CoV-2.

Beside Cas9, other members of the Cas family have also been explored for genomic engineering. Cong et al. reported the development of an enhanced Cas12a for multiplexed precision editing in mammalian cells. The authors showed that this system can be utilized for genome editing of long sequences, which can complement existing genetic tools. In addition, Chuang et al. demonstrated the potential of using CasRx, a member of the Cas13d family, to mediate RNA knockdown to treat retinal degeneration. In this study, the authors demonstrated *in vitro* knockdown of the *VEGFA* mRNA with comparable efficiency to RNAi. This provided a novel approach for VEGF inhibition as a treatment of age-related macular degeneration. Furthermore, the authors developed an adeno-associated virus (AAV) to deliver CasRx and pre-sgRNA in an all-in-one system. Notably, delivery of multiple pre-sgRNA in array format demonstrated better knockdown efficiency compared to single pre-sgRNA. The development of an AAV delivery system would facilitate clinical development of gene therapy, given it has established efficacy and safety profile in the retina.

In summary, this Research Topic provides a collection of research articles that covers a range of topics in genome editing, providing a timely review in the field and highlighting its potential to develop novel therapy for a range of diseases.

